# The Ferret as a Model System for Neocortex Development and Evolution

**DOI:** 10.3389/fcell.2021.661759

**Published:** 2021-04-29

**Authors:** Carlotta Gilardi, Nereo Kalebic

**Affiliations:** ^1^ETH Zürich, Zurich, Switzerland; ^2^Human Technopole, Milan, Italy

**Keywords:** ferret (*Mustela putorius furo*), neocortex, brain development, brain evolution, cortical folding, neural stem/progenitor cells

## Abstract

The neocortex is the largest part of the cerebral cortex and a key structure involved in human behavior and cognition. Comparison of neocortex development across mammals reveals that the proliferative capacity of neural stem and progenitor cells and the length of the neurogenic period are essential for regulating neocortex size and complexity, which in turn are thought to be instrumental for the increased cognitive abilities in humans. The domesticated ferret, *Mustela putorius furo*, is an important animal model in neurodevelopment for its complex postnatal cortical folding, its long period of forebrain development and its accessibility to genetic manipulation *in vivo*. Here, we discuss the molecular, cellular, and histological features that make this small gyrencephalic carnivore a suitable animal model to study the physiological and pathological mechanisms for the development of an expanded neocortex. We particularly focus on the mechanisms of neural stem cell proliferation, neuronal differentiation, cortical folding, visual system development, and neurodevelopmental pathologies. We further discuss the technological advances that have enabled the genetic manipulation of the ferret *in vivo*. Finally, we compare the features of neocortex development in the ferret with those of other model organisms.

## Introduction

The ferret (*Mustela putorius furo*) is a small carnivore, domesticated more than 2000 years ago (Davison et al., 1999). There are three principal reasons that have allowed ferret to become a major model organism in developmental neurobiology. First, the ferret is characterized by an expanded and folded neocortex and a diversity of proliferative neural stem and progenitor cells (Kawasaki, 2014; Fernandez et al., 2016; Kalebic et al., 2019). These features are generally accepted to be fundamental for the evolutionary expansion of the neocortex, which in turn is considered to underlie the increased cognitive abilities of humans (Rakic, 2009; Dehay et al., 2015; Fernandez et al., 2016; Sousa et al., 2017; Kalebic and Huttner, 2020). Hence the ferret is used as an animal model to study the evolutionary expansion of the neocortex and those aspects of human brain development that cannot be modeled in organisms with a small brain, such as rodents.

Second, the ferret is born with an immature brain and many of its neurodevelopmental processes, such as cortical folding (gyrification), protract into the first weeks of postnatal life (Barnette et al., 2009; Sawada and Watanabe, 2012). From a practical side, this makes the ferret a more amenable animal model for the study of such processes, than those organisms in which the same processes happen *in utero*, notably primates. Moreover, as its eyes open only after postnatal day (P) 30, ferret is a suitable model system for studying the early development of the visual system and the role of sensory experience therein (Roy et al., 2018).

Third, the ferret is an established model organism for various human pathological conditions, as it shows similarities not only with the human neurodevelopment, but also with the human immune response (Enkirch and von Messling, 2015). Thus, it has been used to model respiratory and neurological infections, along with neurodevelopmental malformations and brain injury. Finally, ferrets very closely mimic the infection and transmission of SARS-CoV-2, suggesting they could be used as animal models for both the respiratory and neurological aspects of COVID-19 (Kim et al., 2020; Richard et al., 2020; Shi et al., 2020).

The ferret has been used in research for more than 100 years (Yeates, 1911) and it exhibits several features that make it a convenient laboratory animal. These include a large average litter size of eight kits and a fairly short gestation period of 40–42 days (Lindeberg, 2008). Importantly, the ferret is a genetically accessible model system. Its genome has been sequenced (Peng et al., 2014) and several different methods that enable acute genetic manipulation in the embryonic and postnatal brain are available (Borrell, 2010; Kawasaki et al., 2012). Finally, ferret transgenesis has recently been established (Johnson et al., 2018; Yu et al., 2019). In this review we discuss those features of the ferret neurodevelopment that make this animal an important model system for the study of cell biology of neural progenitors, neuronal differentiation and migration, mechanisms underlying gyrification, development of the sensory systems and human pathologies pertinent to the central nervous system.

## Neocortex Development

**FIGURE 1:**
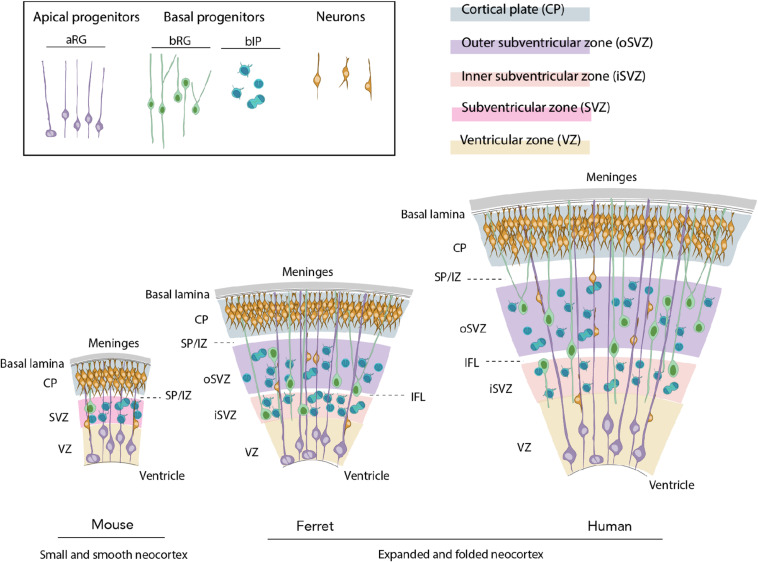
Evolutionary expansion of the neocortex. Ferret and human develop an expanded and folded neocortex, whereas the mouse is characterized by a small and smooth neocortex. The key features of the former are a high abundance of basal progenitors (BP) and particularly basal radial glia (bRG), their morphological heterogeneity, distinction of iSVZ and oSVZ, existence of the inner fiber layer (IFL) and a tangential migration of projection neurons through the germinal zones, intermediate zone (IZ), and subplate (SP) to their final position in the CP.

The mammalian neocortex develops from the dorsolateral part of the telencephalon at the rostral-most part of the neural tube (Rakic and Lombroso, 1998; Taverna et al., 2014). At very early stage of neocortical development, the neuroepithelial cells (NECs) surround the central canal and the cerebral ventricles (Taverna et al., 2014). Once cortical neurogenesis starts, NECs give rise to apical radial glia (aRG), the main type of apical progenitors (Taverna et al., 2014; Figure 1). aRG reside in a dense and highly packed pseudostratified germinal layer, the ventricular zone (VZ) (Figure 1). The asymmetric proliferative division of aRG gives rise to the second major class of progenitor cells, basal progenitors (BPs), that will migrate basally to form the subventricular zone (SVZ) (Taverna et al., 2014; Figure 1). In species with an expanded and folded neocortex, such as ferret and human, the SVZ is further divided into inner SVZ (iSVZ) and outer SVZ (oSVZ), with the latter being particularly rich in highly proliferative BPs (Smart et al., 2002; Dehay et al., 2015; Fernandez et al., 2016; Figure 1). Further basally to the SVZ are the transient intermediate zone (IZ), that will form the white matter, the transient subplate (SP) and the cortical plate (CP) (Figure 1), which will form the six-layered neocortical gray matter, a hallmark of the adult mammalian neocortex.

Newborn projection neurons migrate from the site of their origin in the germinal zones to their final destination in the CP using the radial processes of radial glia (Evsyukova et al., 2013; Buchsbaum and Cappello, 2019; Silva et al., 2019). The mammalian CP is built in an inside-out fashion with the newborn neurons migrating past those already present in the CP to a more superficial (basal) position. Therefore, neocortical layers VI and V, also known as deep layers, are generated first, whereas the layers IV, III, and II, called upper layers, are generated later. The notable exception is the layer I which is situated under the pia and forms first by the Cajal-Retzius pioneer cells (Angevine and Sidman, 1961; Rakic, 1972; Bayer and Altman, 1991; Yamazaki et al., 2004; Molnar et al., 2006; Molyneaux et al., 2007; Franco and Muller, 2013; Lodato and Arlotta, 2015).

## Evolutionary Expansion of the Neocortex

Although the neocortex is present in all mammals, its size has been subjected to evolutionary differences within various mammalian orders (Molnar et al., 2006; Krubitzer, 2007; Rakic, 2009; Kaas, 2013; Kalebic et al., 2017). For example, in mouse the neocortex is small and smooth, whereas in human, macaque and ferret, the neocortex underwent an enlargement. The neocortical expansion was disproportionally greater in the tangential axis, which led to the characteristic cortical folding (gyrification), i.e., formation of gyri and sulci, on the basal side of the tissue (Rakic, 2009; Zilles et al., 2013; Fernandez et al., 2016). Thus, gyrification is thought to come as an evolutionary solution to the outstanding growth of the neocortex and necessity to accommodate additional neurons (Zilles et al., 2013; Borrell, 2018; Kroenke and Bayly, 2018). In addition to the increase in surface area, the neocortical expansion concerns also an increase in thickness (Molnar et al., 2006; Rakic, 2009), which is most pronounced in the neocortical layers II and III (also known as supragranular layers). The thickness of those layers is doubled in primates compared to rodents, with carnivores, such as the ferret, showing intermediate characteristics (Hutsler et al., 2005). Moreover, the thickness of the transient SP compared to the CP thickness is increased in primates versus carnivores and in both primates and carnivores compared to rodents (Kostovic and Rakic, 1990; Molnar et al., 2019; Kostovic, 2020). The ferret SP thickens in regions corresponding to future gyri, whereas it becomes thinner in prospective sulci (Smart and McSherry, 1986a,b). The ferret SP finally disappears when gyri reach their maximum development (Smart and McSherry, 1986a,b). In light of this and the fact that the complexity of SP is increased in species with a folded cortex, it has been suggested that the SP is linked to gyrification (Rana et al., 2019).

An increase in neuronal production is thought to be the key factor underlying the evolutionary expansion of the neocortex. Such increase is a consequence of the increased proliferative capacity of neural progenitor cells and the resulting extension of the neurogenic period (Molnar et al., 2006; Rakic, 2009; Borrell and Reillo, 2012; Dehay et al., 2015; Kalebic et al., 2017; Sousa et al., 2017). Among the neural progenitor cells, BPs are widely considered to be instrumental for the increased neuronal production. In species with a small and smooth neocortex, such as mouse, BPs have a low proliferative capacity, typically dividing only once to give rise to two neurons (Haubensak et al., 2004; Miyata et al., 2004; Noctor et al., 2004; Figure 1). Conversely, they have a higher proliferative capacity and are hence more abundant in species with an expanded and folded neocortex, such as ferret and primates (Fietz et al., 2010; Hansen et al., 2010; Reillo et al., 2011; Betizeau et al., 2013; Kalebic et al., 2019; Figure 1).

Basal progenitors can generally be divided into two subclasses: basal intermediate progenitors (bIPs) and basal radial glia (bRG, also known as outer radial glia or oRG), with the latter being particularly instrumental for the neocortical expansion (Fietz and Huttner, 2011; Lui et al., 2011; Borrell and Reillo, 2012; Figure 1). bRG show a significant difference in their proliferative capacities and morphologies across mammals (Kalebic and Huttner, 2020). In mouse, they are scarce and have a low proliferative capacity, whereas in primates and ferret, bRG are more abundant and have a greater proliferative capacity (Fietz and Huttner, 2011; Lui et al., 2011; Borrell and Reillo, 2012; Kalebic et al., 2017). In terms of morphology, mouse bRG are mainly monopolar and contain a single basal process (Shitamukai et al., 2011; Wang et al., 2011) with only a small fraction of cells exhibiting an apically directed process (Wong et al., 2015; Kalebic et al., 2019; Figure 1). bRG in gyrencephalic species were originally also described as monopolar cells with a single basal process (Fietz et al., 2010; Hansen et al., 2010; Reillo et al., 2011). However, further studies revealed larger heterogeneity with the presence of both bipolar cells and monopolar cells with an apically directed process (Betizeau et al., 2013; Reillo et al., 2017; Kalebic et al., 2019; Figure 1). Importantly, bRG with two basal processes together with a presence or absence of an apically directed process have been reported in human and ferret, but not in mouse developing neocortex (Kalebic et al., 2019; Figure 1). BP morphology has been proposed to be an important underlying factor for the evolutionary expansion of the neocortex (Kalebic and Huttner, 2020) as the progenitors containing more processes are more proliferative and enriched in species with an expanded neocortex (Betizeau et al., 2013; Kalebic et al., 2019).

The prolonged neurogenesis has been suggested to be a consequence of a higher proliferative rate of BPs in animals with an enlarged neocortex. Whereas neurogenesis in mouse lasts ∼9 days, in primates it is 10 times longer and in ferrets it proceeds for ∼5 weeks (Jackson et al., 1989; Noctor et al., 1997; Kornack and Rakic, 1998; Smart et al., 2002; Martinez-Cerdeno et al., 2006). The length of neurogenesis is partially linked to the length of the neural progenitors’ cell cycle, which in mouse is on average 18.5 h (Calegari et al., 2005; Arai et al., 2011), in ferret on average 44 h (Reillo and Borrell, 2012; Turrero Garcia et al., 2015), and in macaque 45 h (Betizeau et al., 2013). A large heterogeneity of ferret neural progenitors is reflected also in the cell cycle duration, with the main difference being the duration of the S-phase (Reillo and Borrell, 2012; Turrero Garcia et al., 2015). The length of the neurogenic period has been proposed to be a particularly important factor of the neocortex expansion within a specific lineage, that is when the neurogenic program is the same (Lewitus et al., 2014). For example, the increase in the length of the neurogenic period has been suggested to be a key factor in the three-fold increase in neocortex size between chimpanzee and human (Lewitus et al., 2014).

## Ferret Neocortex Development

### Onset and Progression of Neurogenesis

**FIGURE 2:**
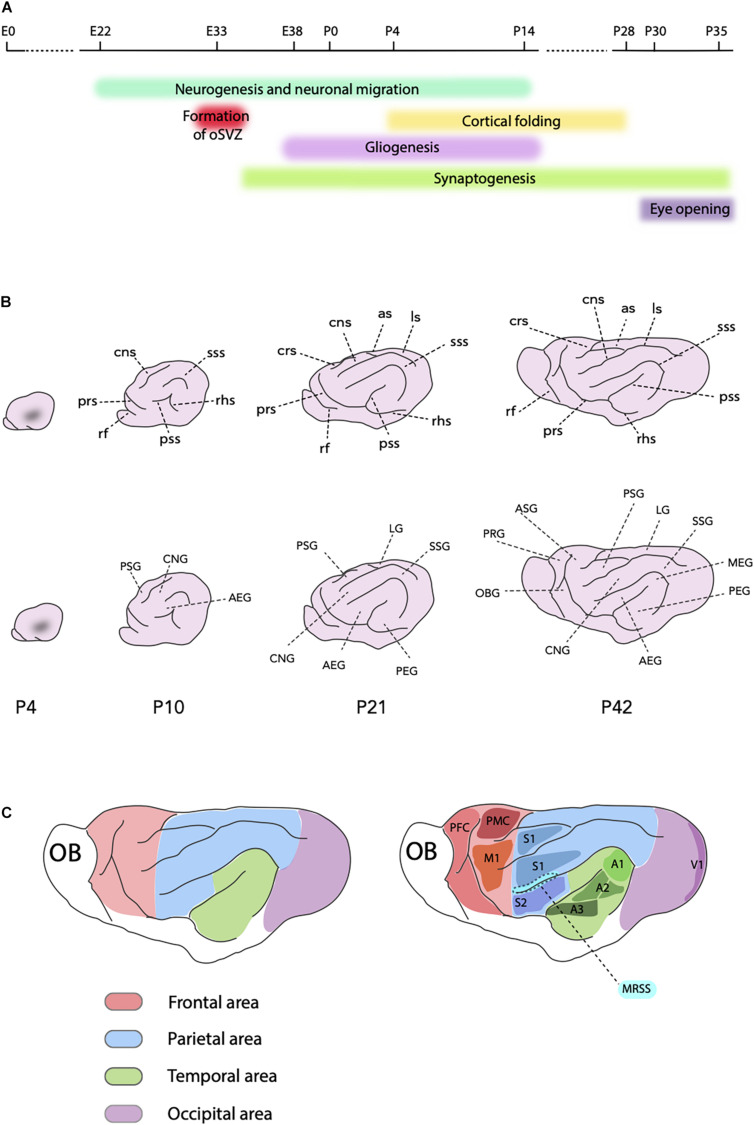
Ferret neocortex development. **(A)** Schematic timeline showing key processes in the ferret neocortex development and the timing of the eye opening. **(B)** Scheme of development of the ferret cortical folding from P4, when the first indentation is visible, until P42, the fully mature stage (on all schemes, rostral is left and dorsal is up). Intermediate P10 and P21 stages are also shown. Upper part shows main sulci (in the clockwise order, P42): as, ansinate sulcus; ls, lateral sulcus; sss, suprasylvian sulcus; pss, pseudosylvian sulcus; rhs, rhinal sulcus; prs, presylvian sulcus; rf, rhinal fissure; crs, cruciate sulcus; cns, coronal sulcus. Lower part shows main gyri (in the clockwise order, P42): PSG, posterior sigmoid gyrus; LG, lateral gyrus; SSG, suprasylvian gyrus; MEG, medial ectosylvian gyrus; PEG, posterior ectosylvian gyrus; AEG, anterior ectosylvian gyrus; CNG, coronal gyrus; OBG, orbital gyrus; PRG, proreal gyrus; ASG, anterior sigmoid gyrus. **(C)** Principal cortical areas in the ferret. Red, frontal area (PFC, prefrontal cortex; PMC, premotor cortex; M1, primary motor cortex); Green, temporal area (A1, primary auditory cortex; A2, secondary auditory cortex; A3, tertiary auditory cortex); Blue, parietal area (S1, primary somatosensory cortex; S2, secondary somatosensory cortex; MRSS, medial rostral suprasylvian area); Violet, occipital area (V1, primary visual cortex or area 17); OB, olfactory bulb.

Ferret SP neurons are generated between the embryonic day (E) 20 and E27 (Jackson et al., 1989; Antonini and Shatz, 1990), whereas the neocortical neurogenesis starts approximately at E22 with the production of neurons of layer VI in the parietal lobe (Noctor et al., 1997; Smart et al., 2002; Barnette et al., 2009; Martinez-Cerdeno et al., 2012; Figure 2A). Compared to rodents, neurogenesis in ferrets starts relatively earlier, which is a distinctive trait of all altricial (born in an underdeveloped state) mammals (Workman et al., 2013). As in other mammals, ferret neocortical neurogenesis proceeds in a rostro-lateral to caudal-medial direction due to the transverse neurogenic gradient. For example, by E29 neurons are already populating the CP in areas rostral to the occipital lobe (McSherry and Smart, 1986), whereas neurogenesis in the prospective visual cortex only starts between E30 and E31 (Jackson et al., 1989). The heterochronicity in generation of upper-layer neurons among different cortical areas can be best appreciated by comparing the data on the prospective somatosensory and visual cortices. In the somatosensory area, the production of neurons belonging to layer IV starts at E33 and continues for 3 days, whereas the generation of supragranular neurons covers the last days of gestation, with very few neurons of layer II produced at P1 (Noctor et al., 1997, 1999). The primary visual cortex (A17 or V1) and the visual association area (A18 or V2) have a thicker layer IV, which is reflected by the layer IV neurogenic period lasting 8 days and ending at P1. Moreover, supragranular neurons in these areas are produced almost exclusively postnatally, finishing at P14 (Jackson et al., 1989).

### Neocortex Expansion: oSvz and Bps

During its embryonic development, the ferret contains all the histological and cell biological features required to build an expanded neocortex (Figure 1). First, it has a thick SVZ which splits into the iSVZ and oSVZ at E33 (Reillo and Borrell, 2012; Martinez-Martinez et al., 2016). Second, it has a high abundance of bRG, the progenitor type most prominently implicated in the neocortical expansion (Reillo et al., 2011; Borrell and Reillo, 2012; Kalebic et al., 2019). Third, it contains all the variety of bRG morphotypes that can be found in human (Kalebic et al., 2019). Collectively, these features enable the development of a folded neocortex with expanded supragranular layers. Similarly to human, ferret BPs comprise roughly 50% bIPs and 50% bRG (Fietz et al., 2010; Kalebic et al., 2019). Like in other studied mammals, ferret neurogenic bIPs express the transcription factor Tbr2 and lack processes during mitosis, whereas ferret proliferative bRG are characterized by the expression of Sox2 and Pax6, by the absence of immunoreactivity for Tbr2, and the maintenance of long radial processes during mitosis (Fietz et al., 2010; Reillo et al., 2011; Kalebic et al., 2018, 2019).

### Gliogenesis

Ferret gliogenesis starts during the last 4 days of the embryonic development (E38–E41) concomitantly with the peak of neurogenesis (Voigt, 1989; Reillo et al., 2011; Figure 2A). Similar overlap of the neurogenic and gliogenic period is characteristic for the neocortex development of other species with an expanded neocortex, notably macaque and human (Zecevic et al., 2005; Rash et al., 2019). However, in species with a small neocortex, such as mouse, these processes are sequential, that is gliogenesis takes place after neurogenesis (Kriegstein and Alvarez-Buylla, 2009). At E38 ferret neural progenitors start expressing the oligodendrocyte transcription factor 2 (Olig2), marker of oligodendroglial progenitors (Reillo et al., 2011). The abundance of Olig2+ cells increases during the first postnatal week and starts decreasing by P14. The astrocytic marker, glial fibrillary acidic protein (GFAP), starts to be detected at P0 and the abundance of GFAP+ cells peaks during the first two postnatal weeks of life (Voigt, 1989; Reillo et al., 2011). It is important to note that in carnivores, GFAP is expressed by both radial glial cells when they switch to astrogliogenic fate and by mature astrocytes (Voigt, 1989; Reillo et al., 2011).

### Neuronal Migration and the End of Neurogenesis

Similar to the onset of neurogenesis, the termination of neuronal production follows rostro-caudal and latero-medial gradients. Whereas in the prospective somatosensory cortex production of neurons is largely completed at birth, in the visual cortex neurogenesis was reported to protract for 2 weeks postnatally (Jackson et al., 1989; Noctor et al., 1997; Figure 2A). Neuronal migration, however, continues postnatally in all brain regions. The majority of prenatally generated neurons reaches their final position in the CP within the first postnatal week, whereas the migration of postnatally generated neurons continues into the second week of life (Jackson et al., 1989; Noctor et al., 1997). This is particularly relevant for the upper-layer neurons, as, for example, in the somatosensory cortex at P1 those neurons are still migrating to their final position (Noctor et al., 1997). Neocortical neurons migrate along the radial processes of radial glia. In lissencephalic species, such as mouse, this migration is radial. In ferret and other gyrencephalic species the neocortical neurons display a tortuous manner of migration which enables tangential dispersion of neurons, that in turn is needed to form the gyri (Gertz and Kriegstein, 2015). At later stages of neocortex development ferret neurons switch from radial to tangential migration, which is accompanied by a sequential use of several different radial glial processes (Gertz and Kriegstein, 2015).

### Synaptogenesis

Synaptogenesis in the ferret starts as soon as migrating neurons reach their final position in the CP (Voigt et al., 1993). Synaptophysin, an indicator of synapse formation, first appears in germinal zones of the prospective visual cortex at E34 (Reillo et al., 2017; Figure 2A). Postnatally, synaptophysin levels reduce in germinal zones and increase in the CP concomitantly with the arrival of migrating neurons, suggesting that synapses are formed in a given layer shortly after the cells reach it. As with other aspects of neocortex development, synaptogenesis is characterized by a rostro-caudal and a latero-medial gradients (Voigt et al., 1993; Herrmann, 1996).

### Cortical Folding and Morphological Maturation

The ferret is born lissencephalic, that is with a smooth brain, and the folding process takes place during the first month of life (Smart and McSherry, 1986a,b; Neal et al., 2007; Barnette et al., 2009; Sawada and Watanabe, 2012; Figures 2A,B). The first visible indentation appears at P4 (Neal et al., 2007; Sawada and Watanabe, 2012; Figure 2B). At this stage, the ferret shows many characteristics of a predominantly immature brain, as the lateral ventricles are still large and the SVZ is quite pronounced. Hence, this stage of ferret brain development is considered to correspond to the period between the gestation weeks (GW) 16 and 20 of the human fetal development, that is when the primary sulci emerge (Lohmann et al., 1999; Barnette et al., 2009). During the next few postnatal weeks of the ferret development, the lateral ventricles shrink, the SVZ becomes less prominent and the CP thickens (Barnette et al., 2009). These features of the morphological maturation of the ferret brain follow both rostro-caudal, and latero-medial gradients. Moreover, they are similar to, but simpler than the patterns seen in the human brain during the second and third trimester of gestation (Barnette et al., 2009; Sawada and Watanabe, 2012). By P14, all main sulci and gyri are formed, but further maturation of the sulcal indentations and gyral folds occurs over the following 2 weeks (Figure 2B). Gyrification is considered completed by P28 (Sawada and Watanabe, 2012). Nevertheless, the shape of the brain afterward changes remarkably, with the rostral part narrowing and elongating (Barnette et al., 2009; Sawada and Watanabe, 2012).

## Cortical Areas

The ferret is an important model organism for studying the development of cortical areas and circuitry. In particular, the ferret gave an important contribution to our understanding of the development and maturation of sensory processing (White and Fitzpatrick, 2007). This is largely due to the fact that the eye opening and the onset of hearing in ferret occur after 1 month of life giving a large postnatal window for studying the sensory development (Lohse et al., 2019). Furthermore, the ferret exhibits certain complex features of brain maturation and neuronal processing that are similar to human and different from rodents. For example, the primary visual cortex in ferrets contains columnar maps of stimulus features, such as orientation selectivity, that are typical of primates (White et al., 2001; White and Fitzpatrick, 2007). In this chapter we outline the contribution of the ferret research to decipher the development of various complex brain functions in the frontal, temporal, parietal and occipital cortical areas.

### Frontal Cortex

Ferret frontal cortex lies in the anterior sigmoid, posterior sigmoid, proreal and the orbital gyri at the most rostral part of the brain (Duque and McCormick, 2010; Fritz et al., 2010; Radtke-Schuller et al., 2020; Figure 2C). Contrary to primate frontal lobe, in the ferret there is no recognizable anatomical boundary on the cortical surface that can delimit motor areas from the rest of the frontal cortex. Furthermore, the ferret lacks a clear granular layer, which is used in primates to define the prefrontal cortex. However, the primary motor cortex has been identified in ferret thanks to the large pyramidal cells in layer V that characterize this area (Radtke-Schuller et al., 2020; Figure 2C). The connectivity and neuroanatomy of the ferret frontal cortex has been increasingly of interest to the scientific community for studies regarding cognitive control of sensory processing and attention as well as goal-directed behavior (Fritz et al., 2010; Sellers et al., 2016; Zhou et al., 2016b). Moreover, a resting-state network in the ferret has been reported to resemble the default mode network in primates (Zhou et al., 2016a), which is often disrupted in neurological disorders, such as schizophrenia, autism spectrum disorders and Alzheimer’s disease (Fox and Greicius, 2010). Hence, this finding raises the possibility that the ferret could be used to model such pathological conditions.

### Temporal Cortex

The auditory cortex of the ferret is located in the anterior, medial and posterior ectosylvian gyri (Kelly et al., 1986a; Kowalski et al., 1995; Bizley et al., 2005; Figure 2C). The ferret has become a widely used animal model for investigating the development and plasticity of auditory processing due to two principal reasons (Lohse et al., 2019). First, the onset of hearing in ferrets occurs only at P32 which facilitates the research of certain developmental processes that in human happen *in utero* (Moore, 1982). Second, ferrets have an audible frequency range that completely overlays with and exceeds the frequency range of humans (Kelly et al., 1986b). Four different tonotopically organized areas, including the primary auditory cortex and the secondary or belt areas, were recognized in the medial and posterior ectosylvian gyri, whereas two fields that are tonotopically not organized were identified in the anterior ectosylvian gyrus (Bizley et al., 2005; Figure 2C). Furthermore, the ferret has become a well-recognized model for studying circuits involved in auditory processing and attention, connecting the primary auditory cortex with the frontal cortex (Fritz et al., 2010). In this context, the rostral ventral posterior auditory field has been identified as a high-order sensory area, homologous to the tertiary (parabelt) area of the primate auditory cortex (Elgueda et al., 2019). Neurons in this area were found to show both changes in auditory responses and encode non-acoustical sound features, such as associated behavioral meaning (Elgueda et al., 2019).

### Parietal Cortex

The primary somatosensory cortex in the ferret is well characterized. The part of the somatosensory cortex involved in body representation lies in the posterior sigmoid gyrus, whereas the part involved in the representation of the face is situated in the coronal gyrus (Leclerc et al., 1993; Rice et al., 1993; McLaughlin et al., 1998; Figure 2C). Secondary somatosensory cortex in the ferret is found in the anterior ectosylvian gyrus (Figure 2C). In addition, the medial suprasylvian sulcus contains a higher order somatosensory area with multisensory neurons that are influenced also by auditory stimuli (Keniston et al., 2009; Figure 2C). The most posterior part of the parietal cortex, involved in visual processing, is located in the lateral and suprasylvian gyri (Manger et al., 2002). The posterior suprasylvian area was found to be crucial for motion perception in the ferret (Philipp et al., 2006). The global motion sensitivity integrates motion information across time and space and it is one of the last visual functions to mature. Recent behavioral and electrophysiological data show a relationship between the posterior suprasylvian area and the ability of ferrets to integrate perceptual motion and form, a high-order visual function which is typically studied in primates (Dunn-Weiss et al., 2019). Hence, ferrets are becoming a suitable model not only for the development of the early visual stages, as discussed in the next section, but also for the visual psychophysics and the study of higher-level visual functions (Dunn-Weiss et al., 2019; Danka Mohammed and Khalil, 2020).

### Occipital Cortex

The visual cortex is located in the occipital lobe and consists of areas 17–21. Area 17 is the primary visual area, also known as the striate cortex, and it contains a thick layer IV (Rockland, 1985; Figure 2C). The border of area 17 with the area 18, the secondary visual area, is marked by a thinning of layer IV and thickening of layer III. The thinning of layer IV accompanied by a decrease in myelination continues further at the boundary between areas 18 and 19 (Innocenti et al., 2002). Ferrets start opening their eyes after P30 and the full opening is reported to occur only at P35 (Christensson and Garwicz, 2005; Figure 2A). Due to the fact that neuronal specialization, synaptogenesis and other events critical for the development of visual circuits occur postnatally, ferrets became an important model organism for visual cortical development (Jackson et al., 1989; Reillo and Borrell, 2012; Danka Mohammed and Khalil, 2020).

Ferrets have thus been used to study various aspects of the maturation of the visual function, in particular the development of orientation selectivity, direction preference and ocular dominance columns (Katz and Crowley, 2002; White and Fitzpatrick, 2007; Roy et al., 2018; Danka Mohammed and Khalil, 2020). Orientation selectivity is one of the first visual functions to appear in mammals and ferret neurons in the primary visual area exhibit it already at the time of eye opening (Chapman and Stryker, 1993; Chapman et al., 1996; White et al., 2001). The majority of ferret neurons in the visual cortex is also characterized by a more vigorous response to a preferred direction of movement of a visual stimulus (Weliky et al., 1996; Li et al., 2006; Van Hooser et al., 2012). This direction selectivity was found to require visual experience (Li et al., 2006), although recent evidence suggests that there is an early phase of instructive plasticity happening before the eye opening (Roy et al., 2020). The work in ferret has thus made a crucial contribution in addressing the fundamental questions regarding how sensory experience shapes the functional development and maturation of a mammalian brain (Roy et al., 2018).

## Techniques for Functional Studies in Ferrets

As the ferret became an established neurodevelopmental model system, different techniques have been developed to manipulate its neocortical development. In this chapter, we review the most commonly used and characterized methodologies along with their respective applications.

### Pharmacological Treatments

**FIGURE 3:**
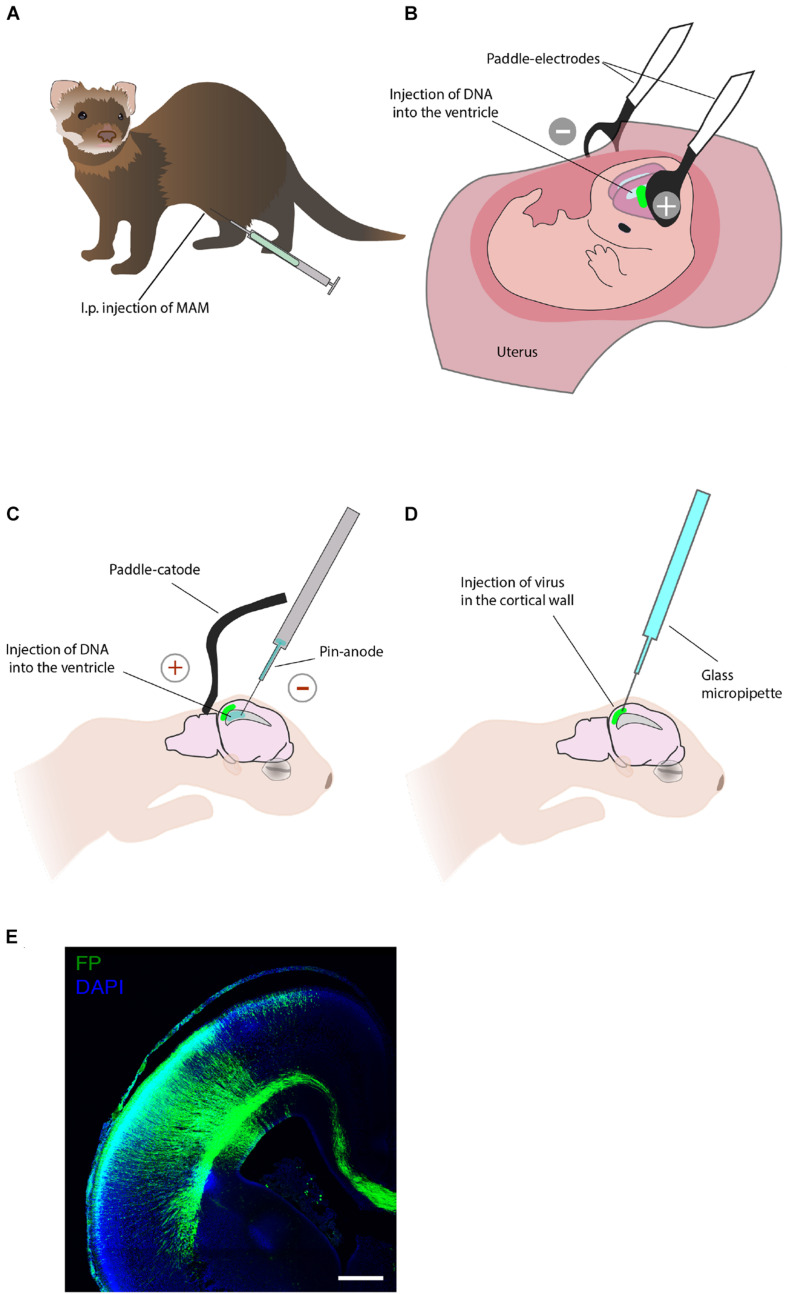
Techniques that allow acute manipulation of the ferret neocortex development *in vivo.* **(A)** Pharmacological treatment. Intraperitoneal (i.p.) injection of the anti-mitotic drug methylazoxymethanol (MAM) allows to study neurogenesis and model cortical dysplasia. **(B)** *In utero* electroporation enables manipulation of gene expression in neural progenitors and their progeny during embryonic development. **(C)** Postnatal electroporation during the first week of postnatal life enables studies of neuronal migration. **(D)** Viral injection at postnatal stages applied to study neural migration. **(E)** An example of the ferret P0 neocortex that was targeted by *in utero* electroporation at E33. Immunofluorescence for GFP (green), combined with DAPI staining (blue). Scale bar, 500 μm. This image has been modified from Kalebic et al. (2018).

Different pharmacological treatments have been explored in ferret to study the neocortex development and model various neuropathological conditions. Noctor et al. (1999) applied to ferret a pharmacological protocol to alter neuronal production at precise timepoints during development. The treatment consisted of an intraperitoneal injection of the anti-mitotic drug methylazoxymethanol (Figure 3A). A single injection of methylazoxymethanol inhibits cell division for 24 h and can hence be used to study the birth date of various neuronal subtypes (Noctor et al., 1999, 2001; Palmer et al., 2001) and to model cortical dysplasia in the ferret (Hasling et al., 2003).

### *In Utero* Electroporation

*In utero* electroporation is a key technique for the acute manipulation of gene expression in the embryonic mammalian brain *in vivo* (Kalebic et al., 2020). This method was first applied to ferrets by Kawasaki and colleagues and it consists of an intraventricular injection of genetic material and subsequent electroporation to permit the entry of delivered molecules to the neuroepithelium (Kawasaki et al., 2012, 2013; Kalebic et al., 2020; Figures 3B,E). *In utero* electroporation has been widely used in ferrets to study the cell biology of neural progenitors (Kalebic et al., 2019; Kostic et al., 2019; Güven et al., 2020; Matsumoto et al., 2020; Xing et al., 2020), histological features of ferrets neurodevelopment (Martinez-Martinez et al., 2016; Saito et al., 2018), and cortical folding (Toda et al., 2016; Matsumoto et al., 2017b; Shinmyo et al., 2017). Of importance for translational research, *in utero* electroporation is increasingly being used to express human-specific genes (Kalebic et al., 2018) and to model human neurodevelopmental pathologies in ferrets (Masuda et al., 2015; Matsumoto et al., 2017a). Furthermore, a recent combination of *in utero* electroporation with the CRISPR/Cas9 technology allows efficient genome editing in the developing ferret neocortex (Shinmyo et al., 2017; Güven et al., 2020; Xing et al., 2020).

### Postnatal Electroporation

Due to the fact that the ferret is born with an immature brain, acute genetic manipulation after birth can be used to study the late stages of the neocortex growth and maturation. Notably, this is not possible in the traditional genetically accessible neurodevelopmental models, mouse and rat, where neurogenesis is completed before birth. The postnatal electroporation protocol, established by Borrell and colleagues, has been used on P1 and P6 ferret kits to label various neocortical cells and to manipulate cortical development (Borrell et al., 2006; Borrell, 2010; Reillo et al., 2011; Figure 3C).

### *In Vivo* Viral Injection

Although viral injection is most often used to study neural structure and function at stages when neocortex development is largely completed (Smith and Fitzpatrick, 2016), it also allows local acute labeling of neural cells embryonically and at early postnatal stages (Figure 3D). Retroviral labeling has been used to analyze cell lineages, clonal dispersion and neuronal migration during ferret embryonic and postnatal development (Reid et al., 1997; Ware et al., 1999; Reillo et al., 2017). Adenoviruses have been largely used to study neuronal migration (Borrell et al., 2006; Gertz and Kriegstein, 2015) and dynamics of neural progenitors (Gertz et al., 2014). Moreover, the viral injection can be combined with postnatal electroporation to achieve double labeling by targeting the VZ cells by electroporation, followed by retroviral infection of the cells residing in the oSVZ (Reillo et al., 2011).

### Transgenic Ferret Lines

Whereas local, acute genetic manipulation methods have been successfully adapted to ferrets and can now be routinely used, there are only a couple of examples reporting the generation of transgenic ferret lines. Johnson et al. (2018) reported the generation of an *Aspm* (abnormal spindle-like microcephaly associated) germline knockout ferret line to model human microcephaly. The knockout was achieved by Transcription Activator Like Effector Nucleases (TALEN)-mediated genome editing. A year later, Yu et al. (2019) reported the creation of a transgenic ferret line expressing a dual-fluorescent Cre-reporter system inserted into the ferret ROSA26 intron 1. In this case CRISPR/Cas9-based genome editing was employed.

## The Ferret as a Model for Human Pathologies

Owing to the emergence of new techniques, notably those that render the ferret a genetically accessible model organism, it is now possible to use ferrets to study the pathophysiology of various diseases. This is most evident when focusing on neurodevelopmental defects and malformations for which other animal models, such as the mouse, have shown to exhibit limitations. The ferret has been used as a laboratory animal since the beginning of the 20th century, firstly to describe in details its embryology (Yeates, 1911). It was soon shown that ferrets can be a suitable model for respiratory diseases (Smith et al., 1933), which resulted in ferrets becoming one of the leading model systems in this field (Enkirch and von Messling, 2015). Although a great part of the biomedical research involving ferrets focuses on neuroscience and respiratory infections, ferrets have also served as a model for gastrointestinal diseases (Hudson et al., 1992), human lung cancer (Aizawa et al., 2013), and filovirus infections, notably ebolavirus (Cross et al., 2016, 2018).

### Respiratory Infections and Syndromes

The ferret is a well-established animal model to study the symptomatology, transmission and immune response related to respiratory viruses, notably influenza viruses (Zhang et al., 2013; Enkirch and von Messling, 2015; Stittelaar et al., 2016). Moreover, ferrets and other mustelids have been used in studies concerning coronaviruses, since this viral subfamily may cause severe acute respiratory syndrome in mustelids (Chu et al., 2008). Very early in the current COVID-19 pandemic it has been shown that ferrets are highly susceptible to SARS-CoV-2 infection and that they display mild symptoms, such as fever, loss of appetite, occasional coughs, and reduced body activity (Kim et al., 2020; Shi et al., 2020). SARS-CoV-2 can be actively transmitted from infected ferrets to naïve ones through direct contact or by airborne transmission even before the peak of the viral RNA copy number has been reached (Kim et al., 2020; Richard et al., 2020; Shi et al., 2020). These characteristics make the ferret one of the animal models that most closely mimics human infection and transmission (Hobbs and Reid, 2020; Mahdy et al., 2020; Munoz-Fontela et al., 2020).

Because the ferret has major similarities in respiratory tract and lung physiology with that of humans it has been long used as a model organism for cystic fibrosis, a genetic disorder caused by mutations in *CFTR* (cystic fibrosis transmembrane conductance regulator) and characterized by chronic infection, inflammation, and mucus obstruction (McCarron et al., 2018). Indeed, *CFTR*-deficient ferrets demonstrated many of the characteristics of human cystic fibrosis disease (Sun et al., 2008, 2010).

### Neurological Infections

Recently, the ferret has been used in research involving infection by Zika virus due to the similarity of both the immune responses and certain neurodevelopmental features with those of human, referred to earlier in this review (Hutchinson et al., 2019). Zika virus is neurotropic and an infection during pregnancy in humans can lead to neurodevelopmental malformations, such as microcephaly, i.e. reduced brain size. In ferret these phenotypes were recapitulated with a high variability among embryos suggesting that the ferret can be used as a valuable model to understand the mechanism of the Zika pathology that can lead to a heterogeneous magnitude of symptoms (Hutchinson et al., 2019).

It is becoming increasingly clear that SARS-CoV-2 has both a short- and long-term impact on the central nervous system and various neurological symptoms have already been described (Caporale and Testa, 2021). It is therefore of great importance to search for a suitable animal model that could successfully recapitulate the key phenotypes found in humans (Sanclemente-Alaman et al., 2020). Considering the value of ferrets to model both the respiratory aspects of COVID-19 and certain complex features of the human neurodevelopment, it is an important animal model to evaluate.

### Introducing Human-Specific Genomic Changes to Model Human Neurodevelopment

Compared to rodents, the ferret shows some of the key neurodevelopmental features similar to humans, notably an expanded neocortex and gyrencephaly. However, certain neurodevelopmental traits are truly human-specific and they are thought to form a basis for our unparalleled cognitive abilities (Rakic, 2009; Sousa et al., 2017; Ardesch et al., 2019; Pattabiraman et al., 2020). Since various human-specific genomic changes in both coding and non-coding regions are underlying such neurodevelopmental traits, a lot of effort has been made to identify the key changes and examine them functionally in different model systems (Silver, 2016; Dennis et al., 2017; Florio et al., 2018; Pattabiraman et al., 2020; Vaid and Huttner, 2020). The ferret is now becoming an important model organism for testing such human-specific genomic changes in the context of an already expanded brain. For example, the addition of the human-specific *ARHGAP11B* to the embryonic ferret resulted in a further expansion of its neocortex (Kalebic et al., 2018). This was best reflected in an increase in cell density in the upper layers of the CP, their radial expansion and the tangential expansion of the entire cortex, which are the key hallmarks of evolutionary expansion of the neocortex (Kalebic et al., 2018).

### Neurodevelopmental Malformations

Neurodevelopmental malformations, such as microcephaly (reduced brain size), lissencephaly (loss of neocortical folding), polymicrogyria (numerous small neocortical folds), dysplasia (abnormal neocortical lamination), and heterotopias (abnormally positioned cells in periventricular or subcortical regions), are linked to various intellectual or motor disabilities (Romero et al., 2018; Klingler et al., 2021).

Primary microcephaly can have various genetic etiologies and can be linked to many syndromal congenital anomalies (Romero et al., 2018). Many attempts have been made to study primary microcephaly in rodent models, however, due to the small size of their brains, rodents could poorly recapitulate some of the phenotypes found in patients. Therefore, primary microcephaly was the first disease modeled in transgenic ferrets by performing the TALEN-mediated KO of *Aspm* (abnormal spindle-like microcephaly associated) (Johnson et al., 2018). The *Aspm^–/–^* ferrets could successfully recapitulate phenotypic features of the human pathology, such as severe microcephaly, with a striking reduction in brain weight and a decrease in brain surface and thickness (Johnson et al., 2018).

Human patients with classical lissencephaly were often found to have mutations in *CDK5* (cyclin-dependent kinase 5) gene (Romero et al., 2018). Studies in *Cdk5* KO mice reported various impairments of the brain development, but the link with lissencephaly remained unexplored (Ohshima et al., 1996; Gilmore et al., 1998). In contrast, when *Cdk5* was acutely knocked out in the ferret developing neocortex via *in utero* electroporation, the size of the gyrus in the electroporated area was smaller and the sulcus was shallower, with respect to the contralateral hemisphere (Shinmyo et al., 2017).

Cortical dysplasia is a neurodevelopmental defect caused by impaired neuronal migration and it is associated with epilepsy and mental retardation. The generation of a ferret model for cortical dysplasia was achieved by injection of the anti-mitotic drug methylazoxymethanol (Noctor et al., 1999, 2001; Hasling et al., 2003). The treatment strongly impaired the generation of layer IV neurons and migration of interneurons from the ganglionic eminence (Noctor et al., 1999; Poluch et al., 2008), which could be alleviated by inhibition of GABAA receptors (Abbah and Juliano, 2014).

Thanatophoric dysplasia (TD) is a severe genetic skeletal dysplasia that results from missense mutations in *FGFR3* (fibroblast growth factor receptor 3), which cause a constitutive activity of the receptor (Pannier et al., 2009). The cerebral cortex of TD patients shows various abnormalities, of which only megalencephaly (abnormally large brain) could be recapitulated in the mouse model (Lin et al., 2003). A ferret model of TD was generated by *in utero* electroporation of FGF8, a ligand of the FGFR3, and exhibited a thickened SVZ and an increased number of BPs (Masuda et al., 2015). Importantly TD ferrets could successfully recapitulate other key abnormalities found in human patients, such as polymicrogyria (Masuda et al., 2015), periventricular nodular heterotopia (Matsumoto et al., 2017a), and leptomeningeal glioneuronal heterotopia (Matsumoto et al., 2018).

### Hydrocephalus

Hydrocephalus is a condition in which excess cerebrospinal fluid accumulates within the ventricles of the brain. In light of the characteristic postnatal protraction of the ferret neurodevelopment, ferret kits at P14 have been used as a model system for the post-hemorrhagic hydrocephalus, which is associated with premature birth (Di Curzio et al., 2013). To induce the hydrocephalus, ferrets received a kaolin (aluminum silicate hydroxide) injection in the cisterna magna, which led to ventricular dilatation, periventricular white matter damage, astrogliosis, corpus callosum atrophy, thus recapitulating typical human hydrocephalus phenotypes (Di Curzio et al., 2013).

### Brain Injury

Ferrets contain a higher white matter content than rodents, similarly to humans (Schwerin et al., 2017). This led to their use as animal models for traumatic brain injury. Using ferrets Lighthall and colleagues developed a method referred to as controlled cortical impact (Lighthall, 1988). This method allows control over biomechanical parameters known to be associated with the traumatic brain injury, such as injury force and velocity and the extent of tissue deformation (Lighthall, 1988; Lighthall et al., 1990; Osier et al., 2015). Recently, controlled cortical impact method in adult ferrets was further optimized and standardized by Schwerin et al. (2017, 2018), generating animal models with transient memory deficits and altered motor skills.

Considering that the degree of maturation of the ferret brain at the moment of birth is similar to that of humans during the last trimester of pregnancy, ferret kits are also used to model preterm and perinatal human brain injury. This model was generated by subjecting the ferret kits to sub-lethal, chronic hypoxia from P10 to P20, which increased astrocytosis and decreased myelination, similar to human patients (Tao et al., 2012).

## Discussion

Here we discuss the possibilities and limitations of using the ferret as a model organism for neurodevelopment. We mainly compare ferrets with the other major mammalian model organisms, notably rodents and primates. We particularly focus on the suitability of ferret as an animal model for brain evolution, brain development and neurodevelopmental pathologies.

### The Ferret as a Model Organism

A plethora of different factors contributes to determine what makes a good model organism. In the past three decades, the mouse has risen as a principal model organism in many biological disciplines, including developmental neurobiology, largely due to its unparalleled genetic accessibility (Ellenbroek and Youn, 2016). However, in the current era of routine next-generation sequencing and genome editing, this advantage is lost and many historic or emerging model organisms are (re)entering the scene. In their recent review, Matthews and Vosshall (2020) list the ten steps required for an organism to become a model organism, giving the example of the mosquito *Aedes aegypti* that has become a neurobiological animal model. In light of those steps, we here discuss the advantages and limitations of using the ferret as a neurodevelopmental model organism.

First, the ferret is a highly suitable and interesting model organism for studying brain evolution, brain development and the related pathologies. As discussed in detail in the following sections, ferrets are more than rodents, but less than primates, similar to humans, in most of the above-mentioned aspects. Second, the ferret has been domesticated for more than 2000 years and used as a laboratory animal for more than a 100 years (Yeates, 1911; Davison et al., 1999). Ferrets are relatively small and, compared to non-human primates, the housing and economic cost of their maintenance are lower. Furthermore, similar to rodents, ferrets have a large average litter size of 8 kits per litter, whereas for most primates the litter size is 1 or 2 (Tardif et al., 2003). Third, the ferret genome has been sequenced (Peng et al., 2014). Although it is still poorly annotated, the first attempts have been made and the first transcriptomic datasets have been produced (De Juan Romero et al., 2015). In other methodological aspects, such as validation of antibody reactivity, the ferret is also lagging behind rodents. Fourth, *in utero* and postnatal electroporations as well as viral injections opened the possibility to introduce genetic manipulation acutely during development (Reid et al., 1997; Borrell et al., 2006; Borrell, 2010; Kawasaki et al., 2012, 2013; Gertz et al., 2014; Kalebic et al., 2020). In this aspect the ferret was the third organism after mouse and rat in which *in utero* electroporation was achieved. Fifth, transgenic ferrets are now available (Johnson et al., 2018; Yu et al., 2019). Moreover [combining the steps 6–9 from Matthews and Vosshall (2020)] genome editing (Shinmyo et al., 2017), routine gene knock-down (Kostic et al., 2019) and knock-out (Güven et al., 2020; Xing et al., 2020), introduction of precise mutation (Yu et al., 2019), optogenetics (Roy et al., 2016) and other genetic tools important for neuroscience have all been introduced to the ferrets. Finally, interesting questions that can be addressed using the ferret as the model organism are not lacking and include, but are not limited to, the considerations we discuss below.

### The Ferret as a Model for the Evolutionary Expansion of the Neocortex

As recently argued by Gilles Laurent, neuroscience has massively benefited from model system diversity and should further embrace comparative and evolutionary approaches in modern brain research (Laurent, 2020). The ferret ideally fits into these considerations by vastly contributing to the evolutionary perspective of the brain. Ferrets and other carnivores diverged from the human lineage earlier than rodents (Bininda-Emonds et al., 2007), yet many of the features of the neocortex expansion are present in the ferret, but not in the mouse. Together with the data collected from other species, this led to a view that the evolutionary expansion of the neocortex happened independently in most mammalian orders, but it has been guided by similar principles (Borrell and Reillo, 2012). This is particularly obvious when looking into neocortical folding, a trait that highly correlates with neocortex expansion (Lewitus et al., 2013; Fernandez et al., 2016). Neocortical folding is present in all mammalian orders (Borrell and Reillo, 2012) and it appears that the Jurassic-era ancestor of all placental mammals already exhibited a folded cortex (O’Leary et al., 2013; Lewitus et al., 2014). In turn this suggests that gyrencephaly was lost and its extent reduced or increased several times independently during mammalian evolution (Kelava et al., 2013).

The enlargement of the neocortex does not concern only its surface area, although this is a dominant trait, but also its thickness (Rakic, 2009; Geschwind and Rakic, 2013). During development, transient SP and germinal zones are thicker in species with an expanded neocortex (Reillo and Borrell, 2012; Dehay et al., 2015; Rana et al., 2019; Kostovic, 2020) and the separation of the oSVZ and iSVZ can be found only in such species (Smart et al., 2002; Dehay et al., 2015). The adult neocortex also exhibits a different thickness, which is on average two-fold greater in primates compared to rodents (Rakic, 2009). This increase is mostly pertinent to the supragranular layers that roughly doubled in primates compared to rodents (Hutsler et al., 2005). Moreover, there is a clear increase in the thickness of the supragranular layers within primates, with humans having 1.5-fold thicker supragranular layers than macaque (Hutsler et al., 2005). Importantly, carnivores exhibit intermediate characteristics and the ferret has supragranular layers that are 2-fold thicker compared to mouse and 2-fold thinner compared to human (Hutsler et al., 2005). The supragranular layers contain neurons that form ipsi- and contra-lateral cortical connections (Lodato and Arlotta, 2015), with the latter thought to play an important role in high-level integrative cortical functions in associative areas (Fame et al., 2011).

Neocortex expansion is characterized by a differential growth of existing neocortical areas as well as the acquisition of novel ones, which in primate brain evolution is particularly relevant in the frontal cortex (Krubitzer and Kaas, 2005; Kaas, 2013). The protomap hypothesis postulates that the final size and pattern of neocortical areas is determined already during development by various molecular gradients that specifically guide and attract afferent systems to appropriate positions where they can interact with specific cells (Rakic, 1988). It has recently been shown that the development of a prospective gyrus vs. a prospective sulcus of the ferret neocortex is also determined by differently expressed genes during embryonic development (De Juan Romero et al., 2015). Interestingly, those differentially expressed genes exhibited a similar expression pattern in fetal human neocortex, but showed no obvious pattern in embryonic mouse neocortex (De Juan Romero et al., 2015).

### The Ferret as a Model for Brain Development

Traditionally the greatest value of the ferret as a model system for brain development came from the characteristic protraction of its brain development into the postnatal period. This allowed researchers to study processes in ferret kits that in human and rodents happen *in utero*. Therefore, thanks to this practical and convenient feature of its neurodevelopment, the ferret rose to one of the principal models for studying cell biology *in vivo* of neural stem cells, neuronal migration and connectivity. For example, the ferret is a good model to address the maturation of the thalamocortical connectivity in an extra-uterine context (Wess et al., 2017; Lohse et al., 2019). It has recently been shown that SP neurons of the ferret auditory cortex respond to sound at very young ages, even before the opening of the ears, suggesting that the early sound experience can activate subplate circuits before permanent thalamocortical circuits are established (Wess et al., 2017).

The second important feature of ferret neurodevelopment is its similarity to the human, especially when looking into neocortex development. This is most obvious when comparing mouse, ferret and human neocortex (Figure 1) development and distinguishing between the quantitative and qualitative differences across these three species. In quantitative terms, the ferret shows intermediate characteristics in various neurodevelopmental aspects. An example brought in the previous section is that the thickness of supragranular layers in the ferret is an exact intermediate between the mouse and human (Hutsler et al., 2005). In qualitative terms, the ferret shares some neurodevelopmental features with humans that are completely lacking in the mouse. Those include, but are not limited to, the expansion of the SVZ and distinction of the oSVZ (Reillo and Borrell, 2012), abundance of BPs and particularly proliferative bRG (Fietz et al., 2010; Reillo et al., 2011; Kalebic et al., 2019), morphological heterogeneity of bRG (Kalebic et al., 2019), tangential dispersion of migrating neurons (Gertz and Kriegstein, 2015), presence of both inner and outer fiber layers (Saito et al., 2018), and neocortical folding (De Juan Romero et al., 2015; Figure 1). Albeit present in both the human and ferret, most of the features mentioned are quantitatively reduced in the ferret. For example, the ferret neocortex contains all the bRG morphotypes seen in the human, but their relative proportions are different as there are less process-rich bRG in the ferret (Kalebic et al., 2019). Moreover, ferret neocortex is folded, but the gyrencephaly index, i.e. a parameter of the extent of cortical folding, is significantly lower than in humans (Lewitus et al., 2014).

During the neocortical neurogenesis the temporal order of neuronal production follows the well-established inside-out rule. Classical research in the ferret has provided important evidence that the potential of neural progenitors to generate lower vs. upper layer neurons is restricted over time (McConnell and Kaznowski, 1991; Frantz and McConnell, 1996; Desai and McConnell, 2000). Upon heterochronic transplantations, early ferret progenitors, that normally produce lower layer neurons, are capable of producing upper layer neurons, whereas late progenitors appear to be more restricted and can only produce upper layer neurons, even when transplanted into an earlier host environment (McConnell and Kaznowski, 1991; Frantz and McConnell, 1996; Desai and McConnell, 2000). Recent transplantation studies of specific subpopulations of neural progenitors in mouse, however, revealed additional complexity (Oberst et al., 2019; Telley et al., 2019). Notably, mouse apical progenitors, that are generally highly proliferative, were found to be temporally plastic and able to re-enter past neurogenic states. In contrast, mouse BPs, that are highly neurogenic, lacked such plasticity (Oberst et al., 2019). In light of this, it would be important to examine if specific subpopulations of ferret BPs, notably the proliferative bRG and neurogenic bIPs, behave differently upon heterochronic transplantation. Furthermore, recent studies identified selected subpopulations of mouse progenitors (Franco et al., 2012; Garcia-Moreno and Molnar, 2015) that selectively contribute to the production of upper layer neurons and that are restricted in its fate potential even before the onset of neurogenesis. Considering the relative increase in upper layer neurons in ferret vs. mouse, it would be useful to examine if subpopulations of such fate-restricted progenitors are enriched in the ferret.

### The Ferret as a Model for Human Neuropathologies

In light of the above-mentioned developmental features, the ferret is considered to be a highly suitable model organism for certain neurodevelopmental pathologies. This primarily refers to neocortical malformations (Klingler et al., 2021), which can be a consequence of impaired progenitor proliferation or neuronal migration (Bizzotto and Francis, 2015; Buchsbaum and Cappello, 2019) and affect neocortex size and shape. Since ferrets exhibit an expanded and folded neocortex, they are suitable for modeling diseases such as microcephaly (Johnson et al., 2018) and lissencephaly (Shinmyo et al., 2017). Considering the important role of ferrets in virology and immunology for modeling human influenzas, it is important to emphasize the role of ferrets for studying the neurotropic effects of viruses such as Zika (Hutchinson et al., 2019) and SARS-CoV-2. Combining the previous application in neurodevelopmental disorders and viral infections, the ferret is becoming an emerging model organism for studying the relationship between the maternal immune activation and neuropsychiatric diseases (Li et al., 2018).

### Limitations of the Ferret as a Model Organism for Neurodevelopment

The ferret is a suitable model for studying some of the mechanisms regulating brain development, but it also exhibits certain limitations. Only some molecular and cellular mechanisms involved in neocortex development and the related pathologies are conserved across mammals and this is particularly relevant for the development of complex traits. For example, neocortical folding is thought to arise through an interplay of genetic and mechanical factors (Borrell, 2018; Kroenke and Bayly, 2018), with some of the genetic underpinnings being fairly conserved between ferret and human (Albert and Huttner, 2015; De Juan Romero and Borrell, 2017). In contrast, an extracellular matrix-mediated mechanism that contributes to folding by modulating tissue stiffness was found to operate in human, but not ferret neocortical tissue (Long et al., 2018).

The protracted postnatal period of brain development has traditionally been one of the greatest reasons for using the ferret kits to model the last trimester of human brain development (Barnette et al., 2009), however, it also poses some limitations. The ferret kit is exposed to environmental sensory stimuli that are not present *in utero*, which can lead to a different development and maturation of cortical sensory areas. An example is the difference in some aspects of maturation of the visual circuitry, which are thought to happen in a hierarchical fashion in humans, but occur in a synchronous manner in ferrets (Danka Mohammed and Khalil, 2020).

Finally, there are ethical considerations to take into account, especially when working with transgenic ferrets. Research involving ferrets is usually subjected to a greater regulatory scrutiny than the one involving mice. In contrast, ferrets could be a suitable replacement for primates when studying developmental mechanisms that are pertinent to all species with an expanded neocortex. Hence, the ethical considerations, along with the space allocation and cost of ferret research, are additional limitations for using ferrets instead of mice, but at the same time provide an advantage for using ferrets as a replacement for primates.

## Conclusion

Ferrets are fascinating creatures whose neurodevelopmental features render them an important model organism for brain evolution, development, and related pathologies. In this review we highlight the key characteristics of the developing ferret neocortex and discuss the tools and techniques that enable us to use these animals for revealing the complexity of the mammalian neocortex development and modeling human neurodevelopmental pathologies.

## Author Contributions

Both authors listed have made a substantial, direct and intellectual contribution to the work, and approved it for publication.

## Conflict of Interest

The authors declare that the research was conducted in the absence of any commercial or financial relationships that could be construed as a potential conflict of interest.
